# Cerebrospinal fluid camk2a levels at baseline predict long-term progression in multiple sclerosis

**DOI:** 10.1186/s12014-023-09418-9

**Published:** 2023-08-29

**Authors:** Dorsa Sohaei, Simon Thebault, Lisa M. Avery, Ihor Batruch, Brian Lam, Wei Xu, Rubah S. Saadeh, Isobel A. Scarisbrick, Eleftherios P. Diamandis, Ioannis Prassas, Mark S. Freedman

**Affiliations:** 1 Department of Laboratory Medicine and Pathobiology, University of Toronto, Toronto, Canada; 2 Department of Medicine, The Ottawa Hospital, 01 Smyth Road, Box 601, Ottawa, ON K1H 8L6 Canada; 3 The Ottawa Hospital Research Institute, Ottawa, Canada; 4 Biostatistics Division, Dalla Lana School of Public Health, University of Toronto, Toronto, Canada; 5 Department of Biostatistics, The Princess Margaret Cancer Centre, University of Toronto, Toronto, Canada; 6 Department of Pathology and Laboratory Medicine, Mount Sinai Hospital, Toronto, Canada; 7 Department of Pathology and Laboratory Medicine, David Geffen School of Medicine, University of California Los Angeles, Los Angeles, United States of America; 8 Department of Physical Medicine and Rehabilitation, Center for Multiple Sclerosis and Autoimmune Neurology, Mayo Clinic, Rochester, MN 55905 USA; 9 Lunenfeld-Tanenbaum Medicine and Pathobiology, University of Toronto, Toronto, Canada; 10 Department of Clinical Biochemistry, University Health Network, Toronto, Canada; 11 Mount Sinai Hospital, Joseph & Wolf Lebovic Ctr, 60 Murray St [Box 32]; Flr 6 - Rm L6-201, Toronto, ON M5T 3L9 Canada; 12 Laboratory Medicine Program, University Health Network, Toronto, Canada

**Keywords:** Multiple sclerosis, Brain-specific proteins, CSF, Biomarkers

## Abstract

**Background:**

Multiple sclerosis (MS) remains a highly unpredictable disease. Many hope that fluid biomarkers may contribute to better stratification of disease, aiding the personalisation of treatment decisions, ultimately improving patient outcomes.

**Objective:**

The objective of this study was to evaluate the predictive value of CSF brain-specific proteins from early in the disease course of MS on long term clinical outcomes.

**Methods:**

In this study, 34 MS patients had their CSF collected and stored within 5 years of disease onset and were then followed clinically for at least 15 years. CSF concentrations of 64 brain-specific proteins were analyzed in the 34 patient CSF, as well as 19 age and sex-matched controls, using a targeted liquid-chromatography tandem mass spectrometry approach.

**Results:**

We identified six CSF brain-specific proteins that significantly differentiated MS from controls (p < 0.05) and nine proteins that could predict disease course over the next decade. CAMK2A emerged as a biomarker candidate that could discriminate between MS and controls and could predict long-term disease progression.

**Conclusion:**

Targeted approaches to identify and quantify biomarkers associated with MS in the CSF may inform on long term MS outcomes. CAMK2A may be one of several candidates, warranting further exploration.

**Supplementary Information:**

The online version contains supplementary material available at 10.1186/s12014-023-09418-9.

## Introduction

Multiple sclerosis (MS) is a neuroinflammatory and neurodegenerative disease of the central nervous system (CNS), resulting in the demyelination of axons and ultimately axonal and neuronal loss. The individual clinical course of MS is highly variable. Patients may experience little to no disease progression for years, whereas others progress rapidly despite attempts at immunosuppressive treatment [1–4]. The number of disease-modifying treatments (DMT) for MS has increased significantly in the past decade. However, selection of the most appropriate therapy initially, and optimisation of therapy in line with clinical response remains a challenge. As such, there is an unmet need for reliable biomarkers that can help prognosticate and monitor disease activity, thereby guiding individualised therapeutic choices.

Cerebrospinal fluid (CSF) is a promising source of biomarkers for diseases of the CNS due to its direct contact with the brain and its ability to reflect biochemical changes related to the underlying disease [5]. Mass spectrometry-based proteomics is a powerful tool for the multiplexed detection and quantification of proteins in biological fluids such as CSF. Specifically, targeted mass-spectrometry allows for the quantification of desired proteins with high sensitivity, specificity, accuracy, and reproducibility. The most widespread technique for protein identification is the shotgun method, where proteins are digested into peptides, separated by chromatography, and measured by a mass spectrometer. This technique can achieve high throughput. A targeted approach like parallel reaction monitoring (PRM), or selected reaction monitoring (SRM) can be used in proteomics for the relative and multiplexed quantification of proteins in complex biological samples. Protein quantification is based on the selection of a predefined precursor ion and its fragmentation pattern.

Our group previously developed a targeted liquid chromatography-tandem mass spectrometry assay (LC/MS/MS) to simultaneously monitor brain-related proteins in CSF samples, with relatively low sample volume requirement. The selection of the proteins for inclusion in this assay was accomplished by experimental shotgun proteomic search of the CSF and mining of the Human Protein Atlas, to identify brain-enriched proteins [6–8]. Although this panel of proteins was identified in healthy controls, many of the most highly enriched proteins included several proteins with inflammatory and structural roles which could serve as markers of inflammation and/or neurodegeneration seen in MS.

Here, we used this in-house, upgraded targeted mass spectrometry assay to evaluate the prognostic value of soluble brain-specific proteins in CSF, measured near MS diagnosis, for prediction of long term (~ 15-year) clinical outcomes.

## Materials and methods

### Study population and sample collection

Fifty-three CSF samples were obtained from the Ottawa MS biobank (Ontario, Canada). This biobank contains samples and clinical data from patients followed at the Ottawa MS clinic since 1994. Clinical data was collected at baseline sampling and each subsequent visit to the MS clinic, including dates of first reported symptom onset, clinical disease subtype (Clinically isolated syndrome or CIS, relapsing remitting MS or RRMS, secondary progressive MS or SPMS, primary progressive MS or PPMS; determined by the 2010 McDonald Criteria [9]) and Expanded Disability Status Scale (EDSS) scores.

Samples were collected under consent at the time of a patient’s diagnostic work-up (within the first 5 years of symptom onset) by lumbar puncture, centrifuged, and stored at − 80 °C in polypropylene tubes. Prior to analysis, aliquots were selected and coded by a laboratory technician, blinding investigators to patient specific details; unblinding occurred only after LC/MS/MS analysis. Collected CSF samples were shipped on dry ice to Mount Sinai Hospital, Toronto, Canada and stored at − 80 °C until processing. All samples were thawed, processed, and assayed as a single batch, to avoid inter-batch variability.

The consented control CSF samples included in this study were collected from individuals during the same time frame as MS patients and were determined to have non-inflammatory neurological ailments (migraine, fibromyalgia, chronic fatigue, bell’s palsy, somatization disorder, microvascular disease, labyrinthitis and conversion disorder). These samples were selected based on age and sex ratio at the time of sampling to match the MS subjects.

**Fig. 1:**
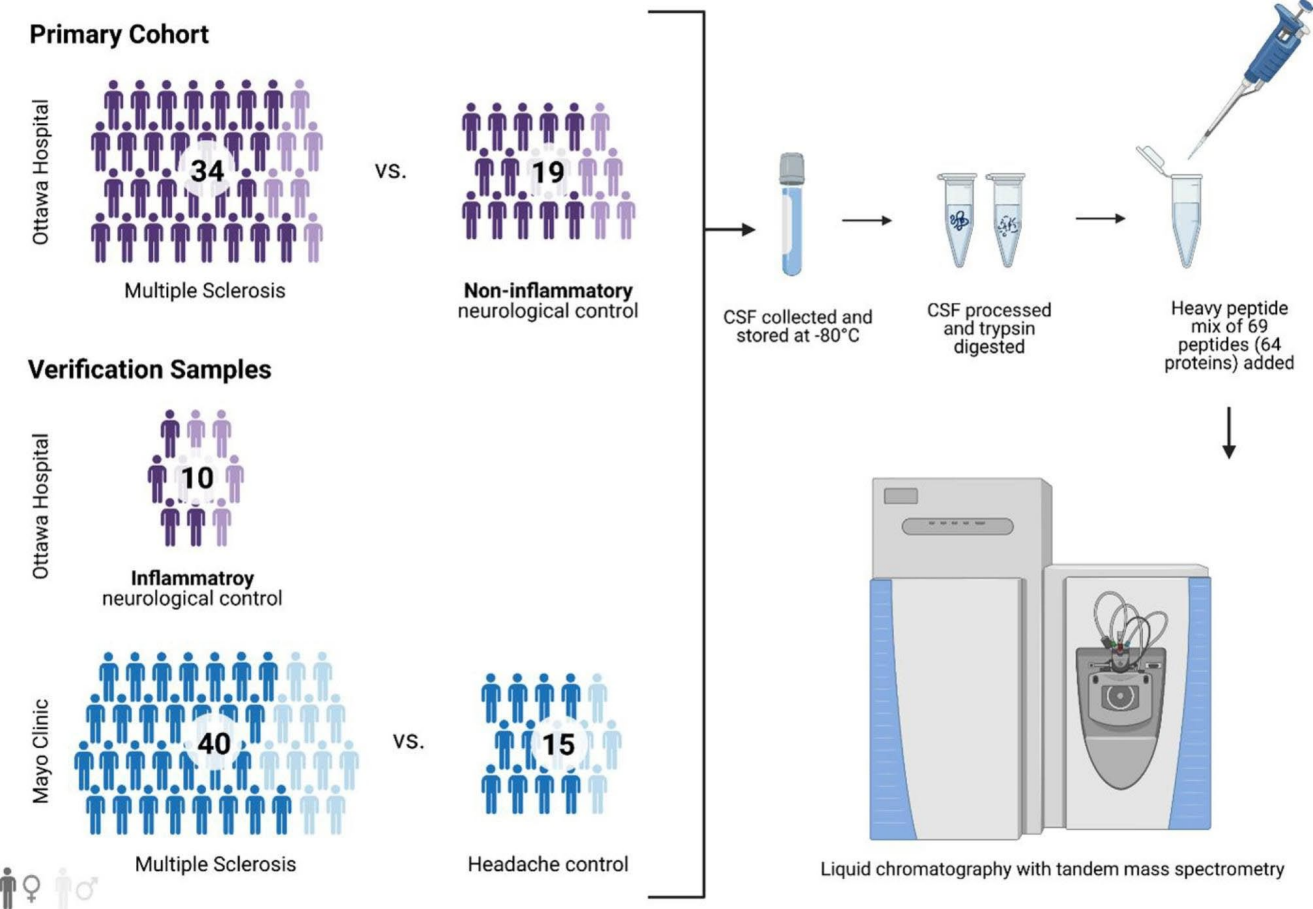
Overview of the study populations and schematic proteomic workflow. The CSF of cohorts comprising MS and control subjects was analyzed. The total number of subjects per cohort group is depicted. Dark and light shades represent female and male subjects, respectively

To further verify any findings from the aforementioned cohort we used non-MS inflammatory neurological control samples (neurosarcoidosis, Guillain-Barre syndrome, Churg-Strauss syndrome, chronic idiopathic demyelinating polyneuropathy, acute disseminated encephalomyelitis and neuromyelitis optica spectrum disorder) from the Ottawa MS biobank, as well as a separate cohort of MS and headache control samples from the Mayo Clinic (Rochester, MN, USA). Study populations are summarized in Fig. 1. Sample processing for these samples was identical to the primary cohort.

Written informed consent had been obtained from all patients prior to inclusion in the Ottawa MS biobank and Mayo Clinic Biobank. Study protocols were approved by the Ottawa Hospital Research Ethics Board (REB # 20,180,518- 02 H), Mount Sinai Research Ethics Board (REB # 19-0321-E), and the Mayo Clinic Institutional Review Board (IRB#:08-007810). All methods and procedures were performed in accordance with the REB recommendations.

### Parallel reaction monitoring assay

A PRM assay was developed for simultaneous quantification of 52 brain-related proteins, as described elsewhere [6, 8]. In addition to these 52-proteins, 12 brain tissue-enriched proteins were later added to the assay to expand the panel. The 64 proteins (69 peptides) quantified in this study are summarized in Supplementary Table 1.

### Mass spectrometry sample preparation

The CSF sample preparation protocol was identical to that employed in our previous work [6, 8]. In brief, each CSF sample was thawed and volumes equivalent to ~ 15 µg of total protein were used for protein digestion. CSF samples were denatured with 0.05% RapiGest (Waters, USA) and reduced with 5 mM dithiothreitol (DTT) (Sigma- Aldrich, Canada) at 60 °C for 40 min. Samples were alkylated with 15 mM iodoacetamide (IAA) (Sigma-Aldrich, Canada) for 60 min in the dark at room temperature. For digestion, trypsin (Sigma-Aldrich, Canada) dissolved in 50 mM ammonium bicarbonate (1:30 trypsin-to-total protein ratio) was added to CSF and left on a shaker overnight at 37 °C. The next day, trifluoroacetic acid (Sigma- Aldrich, Canada) was added to a final concentration of 1% and placed on a shaker at 37 °C for 40 min. The samples were then centrifuged at 13,000 g for 30 min, and the supernatant was kept. A mixture of 68 isotopically labelled peptides (52 candidates from the previous study [6, 8] and C1QTNF4, CDH18, CDH8, GPR158, IGLON5, LGI1, MDGA2, PCDH8, PCDH9, PTPRN, TMEM132A, TMEM59L) were spiked into the digest. Peptides were purified by extraction using OMIX C18 tips (Agilent technologies, USA), eluted in 3 µL of buffer C (64.9% acetonitrile, 35% water, and 0.1% formic acid) and finally diluted with 57 µl of buffer A (0.1% formic acid).

### Liquid-chromatography-tandem mass spectrometry (LC-MS/MS)

Each sample (18 µL) was loaded onto a 0.75 μm ID × 3.3 cm IntegraFrit (New Objective, USA) trap column using the EASY-nLC 1000 system (Thermo Fisher Scientific) running buffer A (0.1% formic acid). Peptides were eluted from the trap column using an increasing concentration of buffer B (99.9% acetonitrile and 0.1% formic acid) onto a 0.75 μm ID × 15 cm analytical PicoFrit column (New Objective, USA) at a flow rate of 300 nL/minute. Trap and analytical columns were packed in-house with 5 and 3 μm Agilent Pursuit C18 media, respectively. The liquid chromatography, EASY-nLC 1000 system (Thermo Fisher Scientific), was coupled online to a Q-Exactive HF-X (Thermo Fisher Scientific) mass spectrometer with a nanoelectrospray ionization source. A 60-minute parallel-reaction monitoring (PRM) method was set up on the Q-Exactive HF-X mass spectrometer (Thermo Fisher Scientific). The full MS1 scan from 355 to 1500 m/z was acquired in the Orbitrap at a resolution of 120,000 (at 200 m/z). Automatic gain control for MS1 was set to 1 × 10^6^ with a maximum injection time of 120 milliseconds. PRM MS/MS spectra were acquired at a resolution of 15,000 (at 200 m/z). Automatic gain control target for MS2 was set to 2 × 10^5^ with a maximum injection time of 120 milliseconds, isolation window of 0.4 m/z and optimized normalized HCD collision energy. MS1 and MS2 spectra were acquired in ‘profile’ mode with 10 ppm inclusion mass accuracy and a scheduled duration of 5 min for each peptide. Blinded clinical samples were analyzed in duplicate.

### Data Analysis

XCalibur software version 4.3.73.11 on Q-Exactive HF-X was used to generate raw files. The raw files were uploaded to Skyline software version 20.1.0.155 which was used for peak integration and quantification of the area under the curve (AUC). Relative quantification for each peptide is reported as the average AUC_light_/AUC_heavy_ (L/H) over 2 technical duplicates. PRM data were manually evaluated, and samples with poor integration and unreliable quantification were excluded.

### Quality Control

A CSF pool was prepared (with 16 individual CSF samples) and digested as a pool, simultaneously with clinical samples. CSF pools were spiked with isotopically labelled peptides. These samples were used for testing assay reproducibility during analysis of clinical samples. The quality control samples were analysed once before the run, every 3 days during the run, and once after the 15-day final run. Reproducibility was assessed by analysing L/H for each peptide during the run sequence. The median coefficient of variation across all LC-MS system stability was also confirmed by running 10 fmol (on column) bovine serum albumin, every 10 runs.

### Statistical analysis

Tertile analysis was performed utilizing GraphPad Prism version 9.0.0 (GraphPad Software, San Diego, CA) and IBM SPSS statistics software (IBM Corp., Armonk, New York USA). Protein distributions were visually inspected and log transformations were applied to those with skewed distributions.

#### Distinguishing controls and MS patients

Mann-Whitney U tests were used to identify proteins with baseline differences between the controls and MS patients. For proteins with differences significant at p < 0.05, unadjusted and age-adjusted logistic regression models were used to predict MS and the area under the receiver operating characteristic (ROC) curve (AUC) was computed using the R statistical programming language [10]. The p-values corresponding to the protein estimates from the logistic regression models were adjusted to control for multiple comparisons.

To explore whether a biomarker signature of multiple proteins could predict MS, two multivariable modelling strategies were used. Method 1 was a forward step-wise regression, beginning with the most statistically significant protein and adding subsequent markers, one at a time, and retaining if fit was improved as assessed with a likelihood ratio test. Method 2 screened proteins with a Mann- Whitney U test, retaining biomarkers with a liberal p-value of < 0.20 (n = 23) then a backwards step-wise regression was performed, removing proteins if model fit was improved. The ability of the resulting groups of biomarkers to distinguish cases and MS patients was then re-evaluated using the independent validation data.

#### Predicting disease progression

Kruskal-Wallis tests were used to identify proteins with baseline differences between controls, MS patients who reached an EDSS score of 4 and those who did not, in the follow-up period. Significance was defined as a p < 0.05 between patients who reached an EDSS score of 4 and those who did not.

To determine if baseline protein levels were associated with change in EDSS score, generalised estimating equations were used to model EDSS score as a function of time and protein tertile, with an interaction term to determine if the rate of change was associated with baseline protein levels. An autocorrelation working covariance structure was used.

Kaplan–Meier survival curves were used to compare the average time to develop EDSS ≥ 4 or to convert to progressive phenotypes (neurologist ascertainment of primary progressive or secondary progressive disease) between the tertiles of protein levels at baseline. Log-rank tests were used for between-group comparisons of the survival curves, using permutations for the reference distribution to account for the small number of observed events.

## Results

### Patient demographic and clinical data

Patient descriptions and relevant clinical data are outlined in Table 1. The baseline demographic details were similar between patients and controls. The mean age for MS patients was 42 (range 24–61), while the mean age in the control group was 35 (range 20–45). The percentage of females in the MS group was 80% (28/34) and for the control group it was 73.7% (14/19). At baseline sampling, the median EDSS score was 1.5 (range 0–7, IQR 0.75). Of the 34 MS patients, 8 were diagnosed with clinically isolated syndrome (CIS), 16 with relapsing remitting MS (RRMS), and 10 with primary progressive MS (PPMS).

**Table 1:** Demographic and clinical information of study participants (Primary cohort)

	Primary Cohort		Verification Cohorts				
	Multiple Sclerosis	Non-Inflammatory Neurological Control	Inflammatory Neurological Controls	Multiple Sclerosis		Headache Control	
Institution	Ottawa Hospital	Ottawa Hospital	Ottawa Hospital	Mayo Clinic		Mayo Clinic	
Participants, n	34	19	10	40		15	
Age at sampling, in years^a^	42 ± 9	35 ± 6	42 ± 15	46 ± 18		46 ± 12	
Sex-female, n (%)	28 (80.0%)	14 (73.7%)	5 (50%)	26 (67%)		12 (80%)	
Initial EDSS^b,c^	1.5 ± 0.75	0	-	1.5 ± 2		-	
Disease course at sampling, n (CIS/RRMS/PPMS)^d^	8/16/10	-	-	0/20/20		-	
Disease course at 10 years, n (CIS/RRMS/SPMS/PPMS)^d,e^	1/17/3/9	-	-	-		-	
Final EDSS ≥ 4, n (%)^e^	13 (43.3%)	-	-	-		-	
Sampling to last follow up, in years^b,e^	14.7 ± 2.5	-	-	-		-	

^a^ Expressed as mean ± SD.

^b^ Expressed as a median ± IQR.

^c^ Expanded Disability Status Scale.

^d^ CIS = clinically isolated syndrome, RRMS = relapsing remitting MS, SPMS = secondary Progressive MS, PPMS = primary progressive MS.

^e^ Four of the thirty-four multiple sclerosis patients did not have long-term follow up and were excluded from these parameters and all long-term clinical outcome analyses.

By the end of follow-up, clinical information was available for 30 patients. As such, 30/34 patients were included in the analyses that involved long-term clinical outcomes. The median EDSS score for the MS patients at the end of follow-up was 3.5 (range 0–10, IQR 4.5). Thirteen of the 30 patients reached an EDSS score of ≥ 4 and 8/30 reached an EDSS score of ≥ 6. On each clinic visit, disease subtype was re-evaluated based on clinical evolution of disease such that by 10 years, only 1/30 patients remained CIS while 17/30 were RRMS,3/30 were SPMS and 9/30 were PPMS.

Patient descriptions for the independent verification experiments are outlined in Table 1. This includes a population of 10 inflammatory neurological controls from the Ottawa MS biobank. These samples were collected at the same institution and during the same timeframe as the MS patients from the primary cohort. Additionally, we included a second, independent cohort of headache controls and MS patients from the Mayo Clinic. These samples were collected from MS and control patients at the Mayo Clinic at first visit (baseline).

### Baseline CSF brain-enriched protein levels in distinguishing MS and controls

**Fig. 2:**
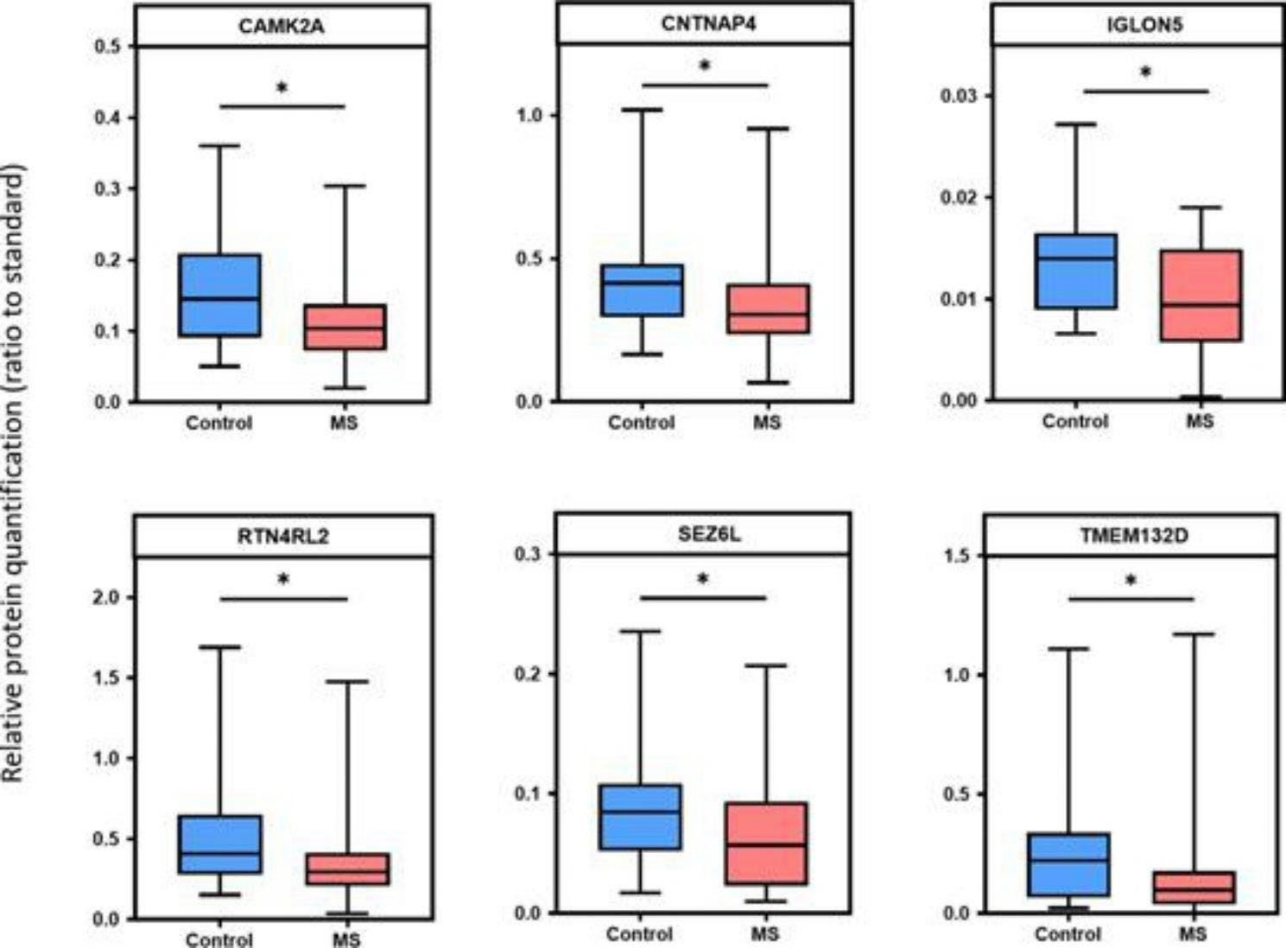
Candidate biomarkers in Ottawa Biobank CSF samples from non-inflammatory controls (n = 19), and MS patients (n = 34) measured using our PRM assay^6^. Relative protein quantification of 6 significant brain-enriched proteins in CSF. Ratio of endogenous protein to isotopically labelled peptides (standard) was lower for MS patients compared to control group (Mann-Whitney U, p-value; CAMK2A p = 0.0476, CNTNAP4 p = 0.0298, IGLON5 p = 0.0434, RTN4RL2 p = 0.0396, SEZ6L p = 0.0396, TMEM132D p = 0.0197). Lines and boxes represent median and interquartile range while bars represent range

When comparing median baseline protein levels between MS and controls, 6 proteins demonstrated a difference that was statistically significantly (Mann-Whitney U, p < 0.05) (Fig. 2) (Mann-Whitney U, p-value; CAMK2A p = 0.0476, CNTNAP4 p = 0.0298, IGLON5 p = 0.0434, RTN4RL2 p = 0.0396, SEZ6L p = 0.0396, TMEM132D p = 0.0197). For each biomarker, a logistic regression model was also fit to determine if the protein was predictive of MS. Initially, an unadjusted model was fit and then age and sex were tested, in that order, and retained if they improved model fit as determined by a likelihood ratio test (Table 2). Age alone was a useful predictor of MS in this cohort and was an important covariate for every biomarker except for GRP158. Sex did not improve model fit and was not retained in any models. No biomarkers were statistically significant after controlling for multiple correction testing (this was true using both Holm-corrected p values and the false discovery rate).

**Table 2:** Area under the ROC curve (AUC) for logistic regression models predicting MS

Protein	Age adjusted ROC AUC (95% CI)		Unadjusted ROC AUC (95% CI)	
	Ottawa Samples	Mayo Samples	Ottawa Samples	Mayo Samples
Age Only Model	0.68 (0.53, 0.82)	0.53 (0.36, 0.69)		
CAMK2A	0.73 (0.60, 0.87)	0.69 (0.53, 0.85)	0.67 (0.51, 0.83)	0.68 (0.53, 0.84)
CNTNAP4_Q9C0A0	0.74 (0.60, 0.88)	0.66 (0.50, 0.83)	0.68 (0.53, 0.83)	0.66 (0.50, 0.82)
IGLON5	0.75 (0.61, 0.88)	0.54 (0.36, 0.72)	0.67 (0.52, 0.82)	0.55 (0.36, 0.73)
RTN4RL2*	0.73 (0.59, 0.86)	0.66 (0.50, 0.82)	0.67 (0.51, 0.83)	0.66 (0.49, 0.82)
SEZ6L	0.73 (0.60, 0.87)	0.55 (0.38, 0.73)	0.67 (0.52, 0.82)	0.56 (0.37, 0.74)
TMEM132D*	0.71 (0.58, 0.85)	0.64 (0.48, 0.79)	0.69 (0.55, 0.84)	0.64 (0.49, 0.79)

*Indicates proteins that were log-transformed prior to analysis

### Multivariate modelling to identify a signature of MS

The forward step-wise regression model to identify a biomarker signature resulted in a three-protein (IGLONS, MOG, SPP1) model with high uncertainty in the parameter estimates and only moderate performance (ROC AUC = 0.80, sensitivity = 58%, specificity = 79%); results were poorer in the validation sample (ROC AUC = 0.74, sensitivity = 60%, specificity = 14%). The second method using backward stepwise regression did not result in any combinations with discriminatory power that could be duplicated in the verification cohort).

### Baseline CSF brain-enriched protein levels and subsequent disease progression

**Fig. 3:**
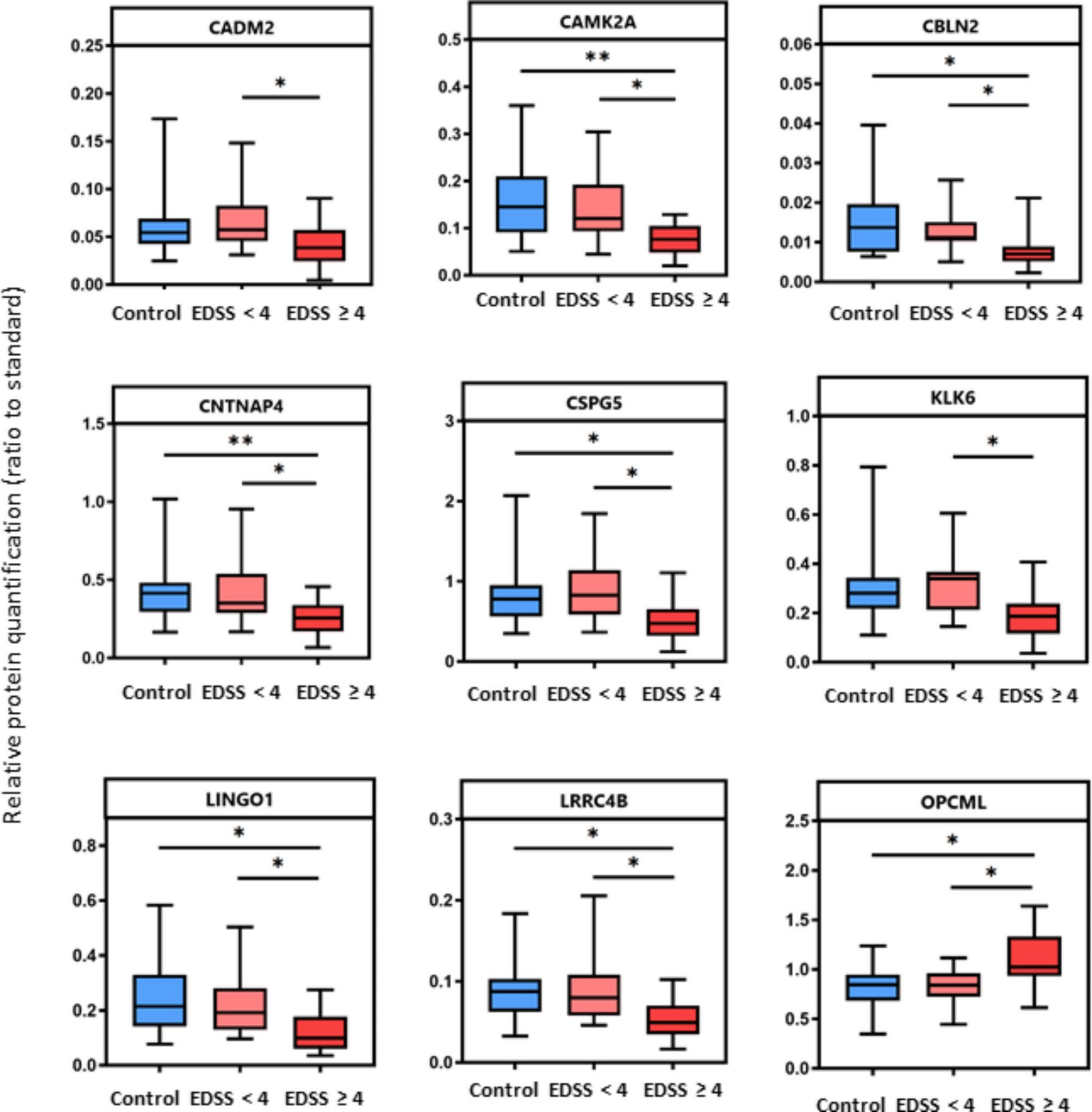
Relative brain-enriched protein quantification and subsequent disease progression. This includes only proteins with statistically significant difference between relative protein quantification of controls, patients who did not reach an EDSS of 4, and patients who did reach an EDSS score of 4 after throughout the follow-up time (Kruskal-Wallis p-values; CADM2 p = 0.027, CAMK2A p = 0.0028, CBLN2 p = 0.0091, CNTNAP4 p = 0.0050, CSPG5 p = 0.0091, KLK6 p = 0.0155, LINGO1 p = 0.0109, LRRC4B p = 0.0122, OPCML p = 0.0109). Lines and boxes depict median and interquartile range, and bars represent total range

As shown in Fig. 3, nine proteins were differentially expressed in patients who reached an EDSS score ≥ 4 versus those who did not, within the 15 years of clinical follow-up. Furthermore, ROC curves were used to compare the ability of the baseline CSF protein levels to predict disease progression (quantified as EDSS ≥ 4 versus EDSS < 4) (data not shown). The most predictive marker (i.e., the marker with the highest ROC AUC) was CAMK2A, followed by CBLN2, LRRC4B, CSPG5, and KLK6 (Supplementary Fig. 1). Furthermore, median baseline CAMK2A levels were significantly different in patients who reached or not an EDSS score ≥ 6 by the end of follow-up (data not shown).

### Baseline CAMK2A levels and subsequent disease progression

**Fig. 4:**
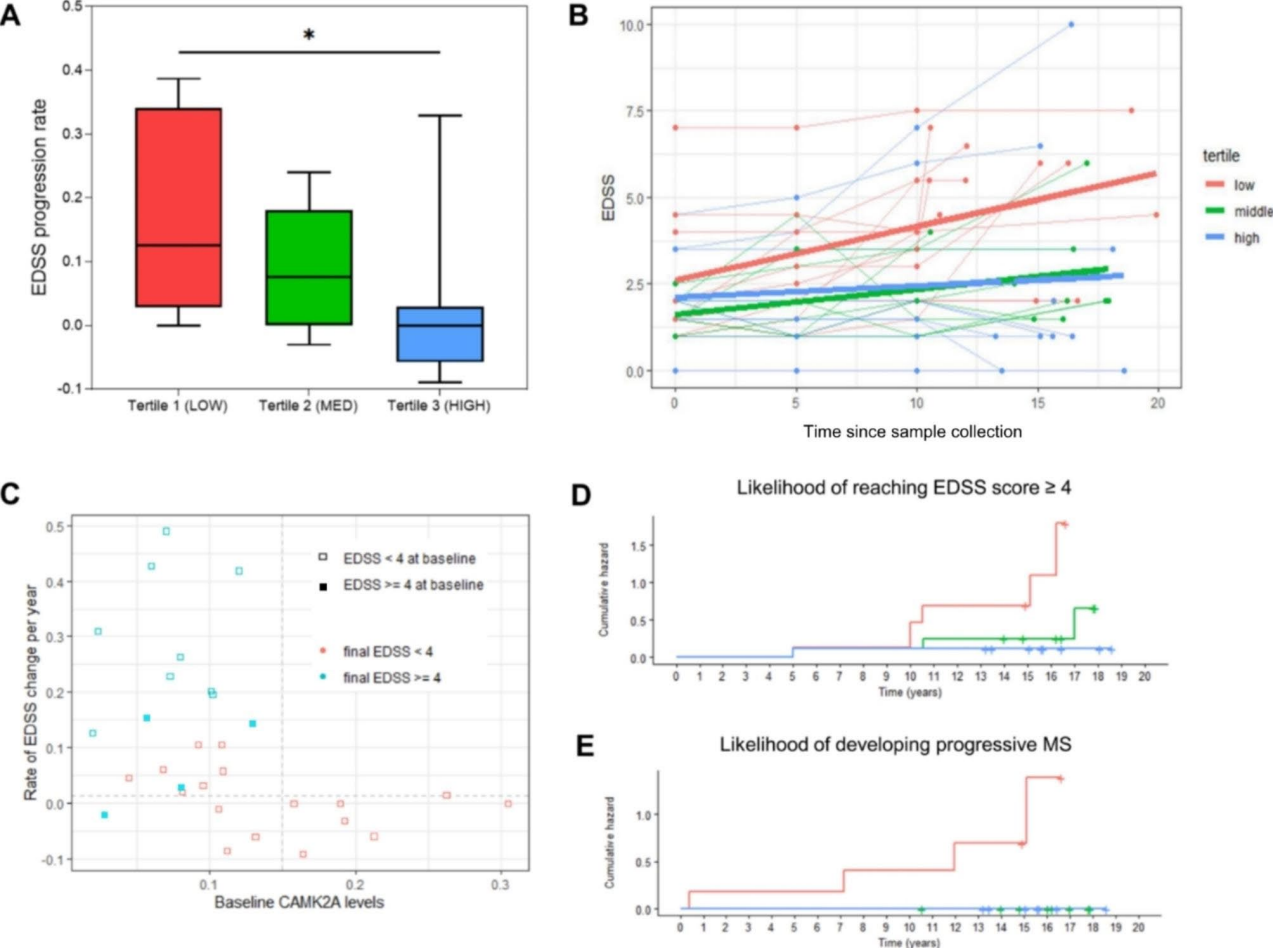
Baseline CAMK2A levels and disease progression. (A) Average rate (median, interquartile range and total range) of EDSS progression (change in EDSS score/year; over time in subgroups of MS patients based on CSF CAMK2A tertiles at baseline (Kruskal-Wallis p = 0.0143*).* (B) Trajectory of EDSS over long-term follow-up time in subgroups of MS patients based on CSF CAMK2A tertiles at baseline (full GEE results in Table 3). (C) Comparison of baseline CAMK2A levels and rate of EDDS change per year. Shading of squares represents baseline EDSS score (Unshaded, baseline EDSS less than 4; Shaded, baseline EDSS greater than or equal to 4). Coloring of squares represents final EDSS score by the end of follow-up time (Red, final EDSS less than 4; Blue, final EDSS greater than or equal to 4). Kaplan-Meier survival curves to compare: (D) hazard of progression to EDSS ≥ 4 (LR chi sq = 8, 2df, p = 0.02) (E) hazard of developing progressive MS (LR chi-sq = 13.1, 2df, p = 0.001) after follow-up in subgroups of MS patients based on CSF CAMK2A tertiles at baseline

One candidate marker, CAMK2A, showed promise as a potential marker of long-term clinical progression, which prompted further investigation. For evaluation of dose-responsiveness, MS patients were divided into three equally sized subgroups based on the tertiles of CAMK2A levels at baseline. As shown in Fig. 4A, patients with the lowest ratio of endogenous protein to standard (1st tertile), had the highest average annual EDSS progression rate, which was significantly higher than the highest tertile. Furthermore, using generalized estimating equations (GEE), we compared the slope of progression in EDSS score over time between the tertiles of baseline CAMK2A (Fig. 4; Table 3). The patients in the 1st tertile progressed with the highest rate, as demonstrated in Fig. 4B.

**Table 3:** Model of EDSS score as a function of time and protein tertile (low, medium high) using generalized estimating equations

Parameter	Estimate	Robust S.E.	95% CI	p-value
Intercept: Low	2.61	0.54	(1.56, 3.67)	< 0.001
Slope: Low	0.15	0.04	(0.08, 0.23)	< 0.001
Tertile Effects				0.11
Intercept: Middle vs. Low	-1.01	0.56	(-2.10, 0.09)	0.07
Intercept: High vs. Low	-0.491	0.66	(-1.79, 0.81)	0.46
Tertile x Time Effects				0.09
Slope: Middle vs. Low	-0.08	0.05	(-0.17, 0.01)	0.09
Slope: High vs. Low	-0.12	0.06	(-0.24, -0.01)	0.04

All thirteen patients who reached an EDSS score of 4 or greater during the follow-up period had a baseline CAMK2A level less than 0.15 (relative quantification as arbitrary units) and patients with baseline CAMK2A levels greater than 0.15 had virtually no EDSS progression (Fig. 4C).

Additionally, we used Kaplan-Meier survival analysis to compare baseline CAMK2A levels with the likelihood of disease progression. As shown in Fig. 4D and E, patients with the lowest CAMK2A levels had the highest risk of reaching EDSS 4 (LR chi sq = 8, 2df, p = 0.02) and highest risk of developing progressive MS, defined as PPMS or SPMS by neurologist assessment (LR chi-sq = 13.1, 2df, p = 0.001).

<Figure4>.

To confirm that the decreased levels of CAMK2A were specific to MS rather than to non-specific neuroinflammation, a population of 10 inflammatory neurological controls identified from the Ottawa MS biobank were used as a comparison (Table 1). There was a significant difference between these control samples and the MS population, suggesting that decreased levels of CAMK2A are likely not attributed to general neuroinflammatory pathways but rather, they are inherent to MS pathophysiology (Supplementary Fig. 2A).

For further verification, baseline CAMK2A levels were measured in a second, independent cohort of headache controls and MS patients (Mayo Cohort). The findings in this verification cohort were consistent with the primary cohort, demonstrating decreased baseline CSF CAMK2A levels in the MS patients compared to the healthy controls (Supplementary Fig. 2B).

## Discussion

In this exploratory analysis, we describe a data-driven proteomic analysis approach to analyze for candidate CSF proteins that could predict long term outcomes in patients with MS. We identified and independently verified an association between low CSF levels of CAMK2A and future disability progression.

Targeted, multiplexed-proteomic technologies are well-recognized for the discovery and validation of CSF biomarkers in different CNS disorders. The advantages of targeted proteomics over other methods such as ELISA, include high specificity and multiplexing capability [11]. The findings from this study (summarized in Suppl. Table 1) support the ability of our PRM assay to measure a large panel of proteins simultaneously and reliably in CSF. The median coefficient of variation (% CV) of the CSF pool run throughout this experiment was 7.7%, where most targeted peptides (> 80%) quantified had CVs less than 30%. PRM assays are often limited by their poor sensitivity for very low abundance proteins in a complex matrix, which likely explains the relatively high CVs for the small minority of peptides quantified in this study.

Here, we evaluated the utility of our in-house developed PRM assay, quantifying 64 brain-related proteins, for discriminating MS and non-inflammatory neurological controls. Additionally, we explored the quantified protein levels and their association to long-term clinical outcomes such as EDSS score and progressive phenotype of MS. We focused on the binary outcome of reaching an EDSS score of 4 (significant disability; ability to walk without aid for 500 m) and 6 (requires a walking aid to walk 100 m). Natural history studies have suggested an EDSS score of 4 as a clinical threshold of irreversible disability. In addition, the binary outcomes offer greater statistical power. The baseline levels of six proteins were identified as potentially useful in distinguishing MS and controls, which included CAMK2A, CNTNAP4, IGLON5, RTN4RL2, SEZ6L and TMEM132D. However, these proteins did not add much predictive ability once controlling for age. The baseline levels of 14 candidate markers were associated with disease progression (EDDS ≥ 4), including the aforementioned six markers. We attempted to combine markers to identify a biomarker signature, however no combination of markers resulted in a discriminatory power that could be duplicated in the verification cohort. CAMK2A emerged as a robust potential prognostic marker of poor clinical outcomes over the follow-up period of more than 15 years.

CAMK2A, calcium/calmodulin-dependent protein kinase type II subunit alpha, mediates calcium-induced signaling which through phosphorylation of various substrates, regulates cellular responses [12]. CAMK2A has not been extensively studied as an MS biomarker, however the limited literature supports the findings in this study. Reduced levels of CAMK2A have been observed in experimental autoimmune encephalomyelitis (EAE), an animal model of multiple sclerosis [13]. Additionally, differences in mRNA and protein CAMK2A levels have been observed in demyelinated human MS hippocampi [14]. In the context of MS treatment, CAMK2A has shown to be important in the pathway for interferon-β (IFNβ) treatment [15, 16]. A genome-wide association study of 337 IFNβ-treated MS patients found enrichment of the CAMK2A gene in IFNβ responders [15]. Furthermore, CAMK2A has been shown to regulate the expression of brain-derived neurotrophic factor (BDNF) through the phosphorylation of cAMP-response element binding protein [17–19]. BDNF has been extensively investigated in MS [20–31]. In general, BDNF shows a neuroprotective role and its lower concentrations have been associated with MS and poorer outcomes [20, 25]. Recent emerging evidence supports the involvement of microRNAs (miRNAs) as key regulators of neuroinflammation neurodegeneration and autoimmune diseases [32]. A specific miRNA, miR-142-3p has shown to be upregulated in animals with EAE and MS patients and levels seem to correlate with prospective MS disease progression [33–37]. The elevated levels of miR-142-3p have been suggested to normalize with MS treatment [38, 39]. Interestingly, the overexpression of miR-142-3p was found to decrease CAMK2A expression and subsequently BDNF expression [19]. The present study thus supports previous findings and offers new insights into the biomarker potential of CAMK2A and possible points of therapeutic intervention. In our sample, CAMK2A was unrelated to age (Pearson ρ = 0.15, p = 0.37), so we do not believe age is driving the association between CAMK2A and EDSS score.

A remarkable observation in this study was that all proteins identified as potential markers had lower abundance in MS patients compared to controls (except for OPCML). It could be expected that CNS proteins have greater abundance in the CSF of MS patients if the higher levels were a result of leakage or release from tissue injury caused by MS disease processes. An alternative explanation is that the proposed markers in this study are functionally relevant in neuro-axonal processes and are essential for normal CNS maintenance and function [40–49]. This would be consistent with the findings of other groups who also observed lower protein abundance in patients compared to controls, both in demyelinating diseases [50–54] and other neurodegenerative diseases like Alzheimer’s disease (AD) or Parkinson’s disease [55–57]. An explanation brought forward by the authors in one of these studies is that the depleted proteins occur because of the breakdown of neural cell membranes in those destined to convert to AD. A similar proposal can be made in MS pathogenesis; disturbed neuro-axonal mechanisms could predispose individuals to undergo a demyelinating event. Alternatively, the MS neuropathological processes may dampen the production of these proteins crucial to CNS integrity, thus lowering their abundance. It is yet to be elucidated if the lower abundance precedes disturbance or is a result of disease pathology.

There are limitations with this study. First, the controls in the primary cohort were non-inflammatory neurological controls (e.g., migraine, fibromyalgia) rather than healthy controls. The strength of these controls is their better resemblance to clinical practice than healthy controls, where patients with neurological symptoms present and have CSF analysed but are ultimately found to not have MS. However, these non-inflammatory controls could present a confounder, as the presence of neurological symptoms in these patients could be reflective of underlying protein alterations. As such, proteins that may have been informative could have been lost due to the lack of significance. The inclusion of age- and sex-matched healthy controls could have helped eliminate this potential confounder, although obtaining CSF on such patients is problematic. Furthermore, the clinical outcomes we used to quantify disease progression were EDSS score and disease phenotype. These clinical parameters were determined by multiple neurologists at different timepoints, which makes them susceptible to inter-rater differences. It should be noted that these are well-validated measures used by MS experts, such as at the Ottawa Hospital and Mayo Clinics [58]. MRI is an important, less subjective covariate that can, to some degree, predict progression early in the disease. Unfortunately, given the long follow-up periods, many of these patients presented in the early 1990s, at which time digitized MRIs were not yet available in the Ottawa Hospital. Given the early time frame of this study (1994–2004), few patients had the opportunity to take contemporary higher efficacy therapies. As such, disease modifying therapy is highly unlikely to be a confounding variable of this study as only 2/34 patients were on DMTs (injectable interferons and glatiramer acetate) at the time of sampling.

In literature, high CSF levels of NFL and GFAP are associated with clinical progression [59]. We did measure NFL and GFAP by the targeted MS assays in this manuscript, however, we did not find these two proteins associated with clinical progression. Although, we did see slight increases in GFAP and NFL in this experiment, they were non-significant differences. We suspect that the heavy labelled isotope that we chose may not be reliably measured, which highlights one of the limitations of our study where we only quantifying a single peptide of these proteins. We performed additional correlation’s and found that within this study, CAMK2A, did correlate with NFL (Spearman r = 0.41, p = 0.0034) and GFAP (r = 0.64, p < 0.0001) measured in this study. Additionally, a previous study on this same cohort measured NFL, GFAP and UCHL-1 using Quanterix SIMOA technology, and these values did correlate with our measurements of CAMK2A (Spearman r = -0.28, p = 0.0498).

We adopted a liberal approach to identifying candidate biomarkers in this study, we conducted an initial screen with a non-parametric test and examined all six proteins that were statistically significant. Subsequent logistic regression, both age adjusted and unadjusted had low predictive ability for these biomarkers (Table 2). Neither forward nor backward stepwise regression models reliably identified a model to predict MS. We feel there is value to this approach when a validation sample is available and the risk of over-fitting a model or falsely identifying predictive proteins is mitigated.

While the association between candidate markers and long-term clinical outcomes presented in this exploratory study are encouraging, we acknowledge the limitations of this group level data involving multiple comparisons. Validating candidates in additional, independent MS cohorts is a critical step for future work. Body fluid biomarkers may one day contribute to determining risk of progression in MS and assist in treatment decisions.

## Conclusions

Collectively, these findings highlight the utility of targeted mass spectrometry-based proteomics to identify markers in the CSF early in the disease, predictive of future MS disease severity. We evaluated the prognostic value of 64 candidate brain-enriched CSF derived proteins for their ability to predict long term clinical outcomes in a cohort followed for at least 15 years. Future studies assessing the candidate markers from this study longitudinally and across additional MS cohorts will be critical in determining the most effective combination of indices that correlate with disease activity. The identification of soluble prognostic biomarkers such as these in early-stage MS could improve patient management by better patient stratification and treatment optimization.

### Electronic supplementary material

Below is the link to the electronic supplementary material.

Supplementary Material 1: **Table 1**. Proteins and Corresponding peptides Monitored in the PRM panel, **Figure 1**. Individual ROC AUC curves for our studied putative markers, **Figure 2**. Decreased baseline CSF CAMK2A levels in the MS patients compared to the healthy controls.

## Data Availability

Consideration will be made by the authors regarding the sharing of anonymized data related to this study.

## Abbreviations

MS: Multiple sclerosis
CSF: Cerebrospinal fluid
CIS: Clinically isolated syndrome
RRMS: Relapsing-remitting multiple sclerosis
PPMS: Primary progressive multiple sclerosis
SPMS: Secondary progressive multiple sclerosis
EDSS: Expanded Disability Status Scale
PRM: Parallel reaction monitoring
LC: Liquid chromatography
CV: Coefficient of variation
FDR: False-discovery rate
DTT: Dithiothreitol
IAA: Iodoacetamide
CAMK2A: Calcium/calmodulin-dependent protein kinase type II subunit alpha
EAE: Experimental autoimmune encephalomyelitis
CNS: Central nervous system
LC/MS/MS: Liquid chromatography-tandem mass spectrometry
