# Supplementary material for: Cerebrospinal fluid camk2a levels at baseline predict long-term progression in multiple sclerosis
Source: Clin Proteomics. 2023 Aug 29;20:33. doi: 10.1186/s12014-023-09418-9 (PMC10466840; doi:10.1186/s12014-023-09418-9)

**SUPPLEMENTARY MATERIAL**

| **Supplementary Table 1**  Proteins and Corresponding Peptides Monitored in the PRM panel | | | | |  |  |
| --- | --- | --- | --- | --- | --- | --- |
| **Accession UniProt** | **Gene Name** | **Protein Description** | **Peptide Sequence** | **Precursor *m/z*** | | |
| P51693 | APLP1 | Amyloid-like protein 1 | DELAPAGTGVSR | 586.80 | | |
| Q96GW7 | BCAN | Brevican core protein | FNVYCFR | 503.23 | | |
| Q8N3J6 | CADM2 | Cell adhesion molecule 2 | SDDGVAVICR | 546.26 | | |
| Q8IUK8 | CBLN2 | Cerebellin-2 | VAFSATR | 376.21 | | |
| Q96KN2 | CNDP1 | Beta-Ala-His dipeptidase | ALEQDLPVNIK | 620.35 | | |
| Q02246 | CNTN2 | Contactin-2 | VTVTPDGTLIIR | 642.88 | | |
| Q8NFT8 | DNER | Delta and Notch-like epidermal growth factor-related receptor | VTATGFQQCSLIDGR | 826.91 | | |
| Q92876 | KLK6 | Kallikrein-6 | LSELIQPLPLER | 704.41 | | |
| O14594 | NCAN | Neurocan core protein | TGFPSPAER | 481.24 | | |
| O95502 | NPTXR | Neuronal pentraxin receptor | VAQLPLSLK | 484.81 | | |
| Q92823 | NRCAM | Neuronal cell adhesion molecule | VFNTPEGVPSAPSSLK | 815.43 | | |
| Q99784 | OLFM1 | Neolin1 | LTGISDPVTVK | 565.33 | | |
| Q14982 | OPCML | Opioid-binding protein/cell adhesion molecule | ITVNYPPYISK | 647.85 | | |
| P23471 | PTPRZ1 | Receptor-type tyrosine-protein phosphatase zeta | AIIDGVESVSR | 573.31 | | |
| P13521 | SCG2 | Secretogranin-2 | ALEYIENLR | 560.80 | | |
| Q9BYH1 | SEZ6L | Seizure 6-like protein | ETGTPIWTSR | 574.29 | | |
| P10451 | SPP1 | Osteopontin | AIPVAQDLNAPSDWDSR | 927.95 | | |
| O15240 | VGF | Neurosecretory protein VGF | FGEGVSSPK | 454.23 | | |
| Q9NT99 | LRRC4B | Leucine-rich repeat-containing protein 4B | DLAEVPASIPVNTR | 741.40 | | |
| Q8N126 | CADM3 | Cell adhesion molecule 3 | LLLHCEGR | 333.18 | | |
| Q8WXD2 | SCG3 | Secretogranin-3 | TEAYLEAIR | 533.28 | | |
| Q15818 | NPTX1 | Neuronal pentraxin-1 | FQLTFPLR | 511.29 | | |
| Q9P0K9 | FRRS1L | DOMON domain-containing protein FRRS1L | HDIDSPPASER | 612.28 | | |
| Q96PX8 | SLITRK1 | SLIT and NTRK-like protein 1 | LSNVQELFLR | 609.84 | | |
| Q96PX8 | SLITRK1 | SLIT and NTRK-like protein 1 | VVCEAPTR | 466.24 | | |
| P61278 | SST | Somatostatin | SANSNPAMAPR | 558.26 | | |
| P61278 | SST | Somatostatin | SANSNPAM[+15.99]APR | 566.26 | | |
| Q16653 | MOG | Myelin-oligodendrocyte glycoprotein | FSDEGGFTCFFR | 735.31 | | |
| P01303 | NPY | Pro-neuropeptide Y | ESTENVPR | 466.23 | | |
| Q86UN3 | RTN4RL2 | Reticulon-4 receptor-like 2 | LFLQNNLIR | 565.84 | | |
| Q99574 | SERPINI1 | Neuroserpin | ALGITEIFIK | 552.84 | | |
| O60241 | BAI2 | Brain-specific angiogenesis inhibitor 2 | LLAPAALAFR | 521.82 | | |
| P14136 | GFAP | Glial fibrillary acidic protein | DNLAQDLATVR | 608.32 | | |
| P23515 | OMG | Oligodendrocyte-myelin glycoprotein | TLDISNNR | 466.74 | | |
| O95196 | CSPG5 | Chondroitin sulfate proteoglycan 5 | EAGSAVEAEELVK | 666.34 | | |
| P09972 | ALDOC | Fructose-bisphosphate aldolase C | ELSDIALR | 458.76 | | |
| P20916 | MAG | Myelin-associated glycoprotein | TQVVHESFQGR | 429.89 | | |
| A6NLU5 | VSTM2B | V-set and transmembrane domain-containing protein 2B | HGPASAANANNAGAASR | 512.91 | | |
| Q9P2S2 | NRXN2 | Neurexin-2 | LGERPPALLGSQGLR | 521.97 | | |
| Q9UQM7 | CAMK2A | Calcium/calmodulin-dependent protein kinase type II subunit alpha | ITQYLDAGGIPR | 652.35 | | |
| Q13554 | CAMK2B | Calcium/calmodulin-dependent protein kinase type II subunit beta | FYFENLLAK | 572.81 | | |
| Q96FE5 | LINGO1 | Leucine-rich repeat and immunoglobulin-like domain-containing nogo receptor-interacting protein 1 | FVAVPEGIPTETR | 708.38 | | |
| Q14C87 | TMEM132D | Transmembrane protein 132D | AIFATAVAQELLQRPK | 586.01 | | |
| Q9UMF0 | ICAM5 | Intercellular adhesion molecule 5 | SGELGAVIEGLLR | 657.37 | | |
| P09104 | ENO2 | Gamma-enolase | YDLDFK | 400.69 | | |
| P14867 | GABRA1 | Gamma-aminobutyric acid receptor subunit alpha-1 | DNTTVFTR | 477.23 | | |
| Q92752 | TNR | Tenascin-R | LNPATEYEISLNSVR | 853.44 | | |
| Q92752 | TNR | Tenascin-R | ITFTPSSGIASEVTVPK | 867.47 | | |
| P02686 | MBP | Myelin basic protein | GVDAQGTLSK | 488.26 | | |
| P07196 | NFL | Neurofilament light polypeptide | EYQDLLNVK | 561.29 | | |
| P20336 | RAB3A | Ras-related protein Rab-3A | LQIWDTAGQER | 658.83 | | |
| Q9C0A0 | CNTNAP4 | Contactin-associated protein-like 4 | LISISGK | 359.23 | | |
| P58417 | NXPH1 | Neurexophilin-1 | YDTPEPYSEQDLWDWLR | 1106.99 | | |
| P58417 | NXPH1 | Neurexophilin-1 | HNSTGQGNVSVSLVPPTK | 911.48 | | |
| P51674 | GPM6A | Neuronal membrane glycoprotein M6-a | EEQELHDIHSTR | 498.57 | | |
| Q9BXJ3 | C1QTNF4 | Complement C1q tumor necrosis factor-related protein 4 | VPGAYFFSFTAGK | 696.35 | | |
| Q13634 | CDH18 | Cadherin-18 | HYQLYVPESAQVGSAVGK | 966.99 | | |
| P55286 | CDH8 | Cadherin-8 | NEDNSLSILAK | 602.31 | | |
| Q5T848 | GPR158 | Probable G-protein coupled receptor 158 | LLGFATVYGTVTLK | 741.93 | | |
| A6NGN9 | IGLON5 | IgLON family member 5 | LGASSASMR | 440.22 | | |
| O95970 | LGI1 | Leucine-rich glioma-inactivated protein 1 | FQELNVQAPR | 601.32 | | |
| Q7Z553 | MDGA2 | MAM domain-containing glycosylphosphatidylinositol anchor protein 2 | GVEIYEPFFTQGETK | 872.92 | | |
| O95206 | PCDH8 | Protocadherin-8 | TVGLQSVYEK | 562.30 | | |
| Q9HC56 | PCDH9 | Protocadherin-9 | DLNISHINAATGTSASLVYR | 701.69 | | |
| Q16849 | PTPRN | Receptor-type tyrosine-protein phosphatase-like N | SDYINASPIIEHDPR | 576.28 | | |
| Q24JP5 | TMEM132A | Transmembrane protein 132A | LTVWAPLLPLR | 639.90 | | |
| Q9UK28 | TMEM59L | Transmembrane protein 59-like | FQPLTLEQHK | 414.23 | | |

Supplementary figure 1
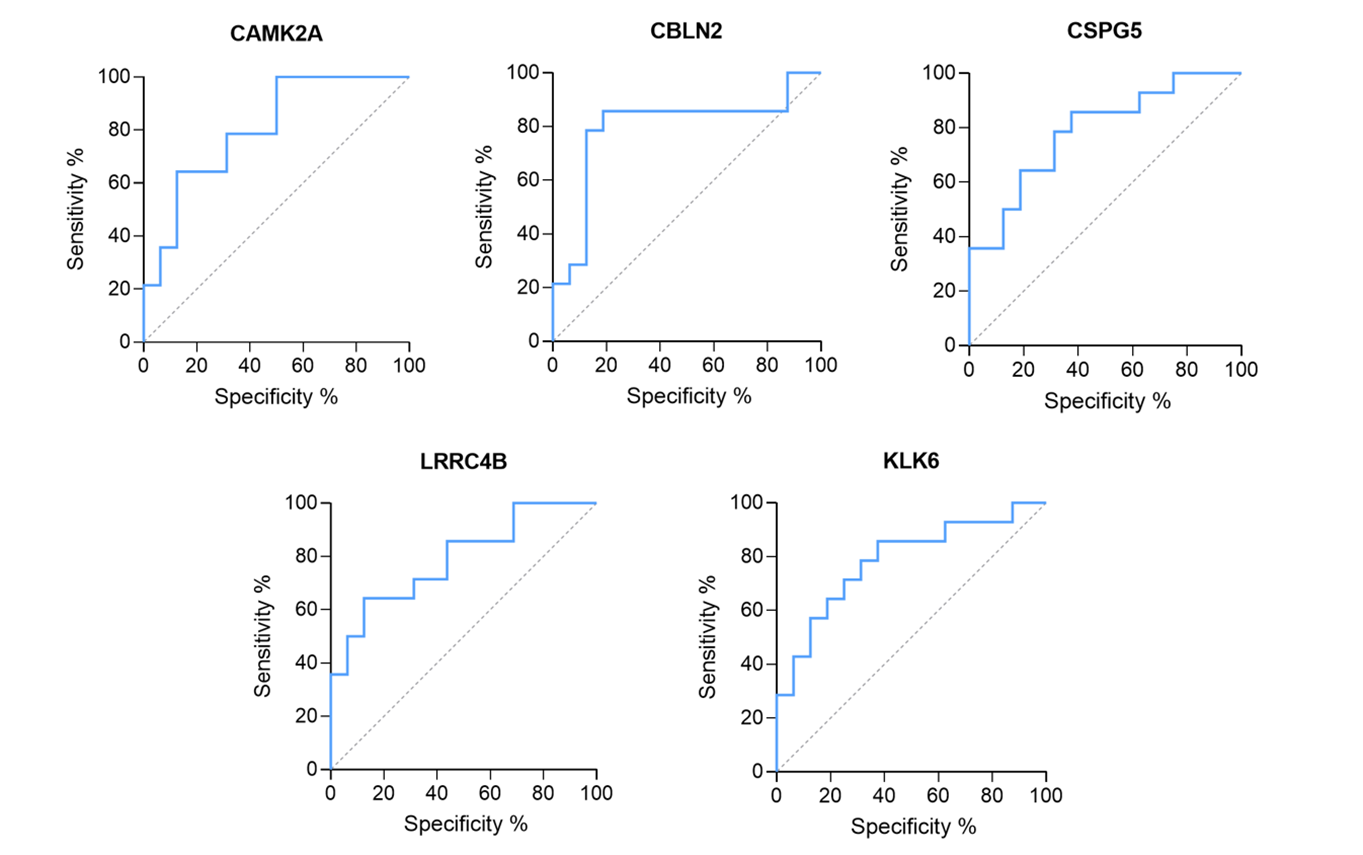


Supplementary figure 2


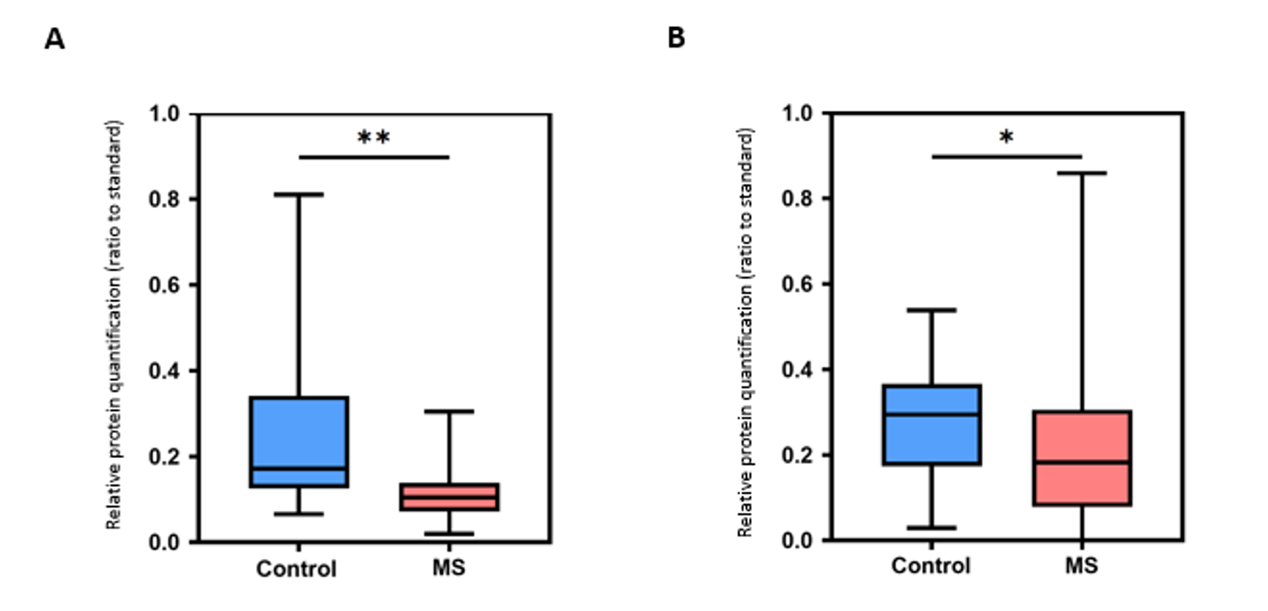

Supplement: Supplementary file 1 — Supplementary Material 1: Table 1. Proteins and Corresponding peptides Monitored in the PRM panel, Figure 1. Individual ROC AUC curves for our studied putative markers, Figure 2. Decreased baseline CSF CAMK2A levels in the MS patients compared to the healthy controls. [file 12014_2023_9418_MOESM1_ESM.docx]
